# Validation of a Prognostic Score to Identify Hospitalized Patients with COVID-19 at Increased Risk for Bleeding

**DOI:** 10.3390/v13112278

**Published:** 2021-11-15

**Authors:** Pablo Demelo-Rodriguez, Francisco Galeano-Valle, Lucía Ordieres-Ortega, Carmine Siniscalchi, Mar Martín Del Pozo, Ángeles Fidalgo, Aída Gil-Díaz, José Luis Lobo, Cristina De Ancos, Manuel Monreal, For the RIETE-Bleeding Investigators

**Affiliations:** 1 Department of Internal Medicine, Hospital General Universitario Gregorio Marañón, Doctor Esquerdo 46, 28006 Madrid, Spain; pbdemelo@hotmail.com (P.D.-R.); lucia.oomere@gmail.com (L.O.-O.); 2 Department of Angiology, Azienda Ospedaliera Universitaria, 43126 Parma, Italy; csiniscalchi@ao.pr.it; 3 Department of Internal Medicine, Hospital Universitario Infanta Sofía, 28703 Madrid, Spain; mar.martin.pozo@googlemail.com; 4 Department of Internal Medicine, Hospital Universitario de Salamanca, 37007 Salamanca, Spain; angelesfidalgo@gmail.com; 5 Department of Internal Medicine, Hospital Universitario de Gran Canaria Dr. Negrín, 35010 Las Palmas de Gran Canaria, Spain; gil_diaz_aida@hotmail.com; 6 Department of Pneumonology, Hospital Universitario Araba, 01009 Álava, Spain; joseluis.loboberistain@osakidetza.eus; 7 Department of Internal Medicine, Hospital Universitario de Fuenlabrada, 28943 Madrid, Spain; cristina.ancos@salud.madrid.org; 8 Department of Internal Medicine, Hospital Germans Trias i Pujol, 08916 Badalona, Spain; mmonreal.germanstrias@gencat.cat

**Keywords:** anticoagulants, COVID-19, VTE prophylaxis, bleeding risk, prognosis

## Abstract

Introduction: Hospitalized patients with COVID-19 are at increased risk for venous thromboembolism (VTE), but also for bleeding. We previously derived a prognostic score including four variables (elevated D-dimer, elevated ferritin, critical illness, and therapeutic-dose anticoagulation) that identified those at increased risk for major bleeding. Methods: We aimed to validate the score in a subsequent cohort of hospitalized patients with COVID-19 receiving standard-, intermediate- or therapeutic doses of VTE prophylaxis. We evaluated its capacity to predict major bleeding, non-major bleeding, and bleeding-related death. Results: The cohort included 972 patients from 29 hospitals, of whom 280 (29%) received standard-; 412 (42%) intermediate-, 157 (16%) therapeutic doses of VTE prophylaxis and 123 (13%) other drugs. Median duration of prophylaxis was 14.7 ± 10.3 days. Major bleeding occurred in 65 patients (6.7%) and non-major bleeding in 67 (6.9%). Thirty patients with major bleeding (46%) died within the first 30 days after bleeding. The prognostic score identified 203 patients (21%) at very low risk, 285 (29%) at low risk, 263 (27%) intermediate-risk and 221 (23%) at high risk for bleeding. Major bleeding occurred in 1.0%, 2.1%, 8.7% and 15.4% of the patients, respectively. Non-major bleeding occurred in 0.5%, 3.5%, 9.5% and 14.2%, respectively. The c-statistics was: 0.74 (95% confidence intervals [CI]: 0.68–0.79) for major bleeding, 0.73 (95% CI: 0.67–0.78) for non-major bleeding and 0.82 (95% CI: 0.76–0.87) for bleeding-related death. Conclusions: In hospitalized patients with COVID-19, we validated that a prognostic score including 4 easily available items may identify those at increased risk for bleeding.

## 1. Introduction

Hospitalized patients with coronavirus disease of 2019 (COVID-19) are at increased risk for venous thromboembolism (VTE) but also for bleeding complications [1,2,3]. In a recent meta-analysis, the overall incidence of VTE in hospitalized patients with COVID-19 was estimated at 17% (95% confidence intervals [CI]: 13.4–20.9) and major bleeding at 3.9% (95% CI: 1.2–7.9) [4]. Current guidelines of antithrombotic therapy recommend the use of VTE prophylaxis for all hospitalized patients with COVID-19, but the optimal dose of prophylaxis has not been clearly established [5,6,7].

Several ongoing clinical trials aimed to compare different dose regimens of anticoagulant interventions in these patients [8,9]. Recently, an international, multiplatform, randomized clinical trial combining data from three trials (ACTIV-4a, REMAP-CAP and ATTACC) compared the use of therapeutic-dose heparin vs. standard prophylaxis in hospitalized patients with COVID-19 [10,11]. Among those receiving therapeutic doses, major bleeding occurred in 3.8% of critically ill patients and in 1.9% of non-critically ill patients. Among patients receiving prophylactic doses, major bleeding occurred in 2.3% and 0.9%, respectively. Another trial (ACTION) reported an incidence of major or clinically relevant bleeding of 8% in patients assigned to therapeutic anticoagulation and 2% in those assigned to prophylactic doses [12]. The risk of death after major bleeding in COVID-19 patients has not been clearly established.

The RIETE (Registro Informatizado de Enfermedad TromboEmbólica) registry is an ongoing, multicenter, international, observational registry of consecutive patients with objectively confirmed acute VTE (ClinicalTrials.gov identifier: NCT02832245) [13]. Since 25 March 2020, the Steering Committee of RIETE agreed to take advantage of the existing platform of RIETE investigators to build a new registry of hospitalized patients with COVID-19 aimed to identify those at increased risk for bleeding, named the RIETE-BLEEDING registry. Using this database, we previously reported that hospitalized patients with COVID-19 receiving intermediate- or therapeutic doses of anticoagulants for VTE prophylaxis had a 5.7% incidence of major bleeding events during admission, and that 45% of these patients died within the first 30 days [14]. Moreover, we built a prognostic score assigning one point each to 4 variables obtained at baseline that were associated with an increased risk for major bleeding: elevated D-dimer levels, elevated ferritin levels, critical illness and therapeutic-intensity anticoagulation. In the current study, we aimed to validate the prognostic score on a subsequent population of hospitalized patients for COVID-19 receiving standard-, intermediate-, or therapeutic doses of anticoagulants.

## 2. Methods

### 2.1. Patients

The previous study included 1965 patients recruited from 25 March to 22 July 2020 in the RIETE-BLEEDING registry. This registry enrolled consecutive patients hospitalized for COVID-19 infection and receiving intermediate- or therapeutic doses of thromboprophylaxis. For the current study, we modified the inclusion criteria and also accepted patients receiving standard doses of prophylaxis, or other drugs (such as vitamin K antagonists or unfractionated heparin). We used data from the period between 23 July and 12 October 2020, when the COVID-19 vaccines were not developed yet. Patients with known acute VTE were excluded from the study. The data were gathered from 32 centers located in 3 countries (Spain, Italy and the United States). Investigators registered data on the clinical characteristics, laboratory levels at baseline, VTE prophylaxis and clinical outcomes occurring during hospitalization, with no intervention planned.

### 2.2. Study Design

Patients receiving at least 3 days of VTE prophylaxis were eligible. In the current study, patients were included irrespective of the doses of thromboprophylaxis. In patients that changed regimens during admission, the regimen that persisted for longer until completion of admission, mortality, or bleeding outcomes were considered. The investigators excluded the patients with prior VTE receiving long-term anticoagulation.

The primary and secondary endpoints were major bleeding and any clinically relevant non-major bleeding, respectively. The RIETE registry defines major bleeding as any overt bleeding event that required a transfusion of ≥2 units of packed red blood cells, or were fatal or located in retroperitoneal, spinal, intracranial, intrathecal, intrapericardial or intraocular spaces [13]. Non-major bleeding events are defined in RIETE as any overt bleeds not meeting criteria for major bleeding but requiring medical assistance. These definitions closely resemble those from the International Society of Thrombosis and Haemostasis.

According to the previously developed RIETE-BLEEDING score, patients were assigned one point for each of the following items obtained at baseline: D-dimer levels >10 times the upper normal range, ferritin levels >500 ng/mL, critical illness (admission in the intensive care unit) and use of therapeutic-intensity anticoagulation. Then, patients were classified as having a very low risk (0 points), low risk (1 point), intermediate risk (2 points) or high risk (3–4 points) for major bleeding.

### 2.3. Variables of Interest

Key data elements included into the database were: demographics, use of mechanical ventilation, recent (<30 days before) major bleeding, concomitant therapy with antiplatelets, site of hospitalization (acute ward vs. intensive care), laboratory tests at baseline (hemoglobin, platelet count, prothrombin time, fibrinogen levels, D-dimer, interleukine-6, ferritin, creatinine clearance), use of thromboprophylaxis (drugs, doses, duration), presence of bleeding during the course of thromboprophylaxis and 30-day all-cause mortality.

Since the different assays for D-dimer levels use different detection antibodies, methods and/or calibrators, and this may lead to confusion [13,14], we compared levels across centers based on times above the upper normal limit. Therapeutic-dose prophylaxis included enoxaparin 1 mg/kg twice daily or 1.5 mg/kg once daily or equivalent doses of other low-molecular-weight heparins, or therapeutic doses of the direct oral anticoagulants. Intermediate-dose prophylaxis was defined as weight adjusted, double-dose prophylaxis, or any dosage greater than the standard dose and lower than the therapeutic-dose. Patients receiving unfractionated heparin or vitamin K antagonists were separately analyzed (“other drugs”).

### 2.4. Statistical Analysis

The study reported categorical data as proportions and continuous data as mean and standard deviation (SD) or median (inter-quartile range [IQR]) days. We used unpaired two-tailed *t*-tests or the Mann–Whitney U test (for those variables found not to follow a normal distribution) for comparisons in the distributions of continuous variables, and chi-squared or Fisher’s exact tests to compare the categorical data between the two groups. We compared demographics, patients’ disposition status (hospitalized in a medical ward or in an intensive care unit [ICU]), blood tests and pharmacological VTE prophylaxis according to the occurrence of bleeding complications. Receiver operating characteristics (ROC) curves were generated to determine the accuracy of the score to predict the study outcomes. ROC curves of models were compared with Hanley and McNeil method. Statistical analyses were conducted with IBM SPSS Statistics (version 25).

## 3. Results

A total of 972 patients hospitalized for COVID-19 in 29 hospitals were included in the study (Supplementary Table S1). Most patients (63%) were men, 48% required ICU admission, and 24% died during admission. Overall, 280 patients (29%) received standard-; 412 (42%) intermediate-, 157 (16%) therapeutic- doses of VTE prophylaxis and 123 (13%) received other drugs (Supplementary Table S2). Median duration of VTE prophylaxis was 14.7 ± 10.3 days.

Compared to patients on standard doses, those receiving therapeutic doses of anticoagulants were most likely to be men, younger, to weigh more and to have higher levels of D-dimer or creatinine clearance at baseline (Table 1). During the course of VTE prophylaxis, 65 patients (6.7%) suffered major bleeding (in the gastrointestinal tract 28%, orotracheal 22%, hematoma 15%) and 67 (6.9%) had non-major bleeding (orothracheal 22%, gastrointestinal 10%), as shown in Table 2. The proportion of patients with major bleeding did not significantly vary according to the intensity of VTE prophylaxis.

Compared to patients that did not bleed, those with major bleeding were more likely to be admitted in an ICU, to have higher rates of D-dimer or ferritin, or to be using antiplatelets concomitantly (Table 3). Patients with non-major bleeding were also more likely to be in an ICU, or to have anemia or higher levels of D-dimer or ferritin at baseline than those who did not bled. Thirty of the 65 patients (46%) with major bleeding, and 22 patients (33%) with non-major bleeding died within the first 30 days after bleeding. Among 840 patients that did not bleed, 178 (21%) died during the first 30 days of hospital stay.

**Figure 1:**
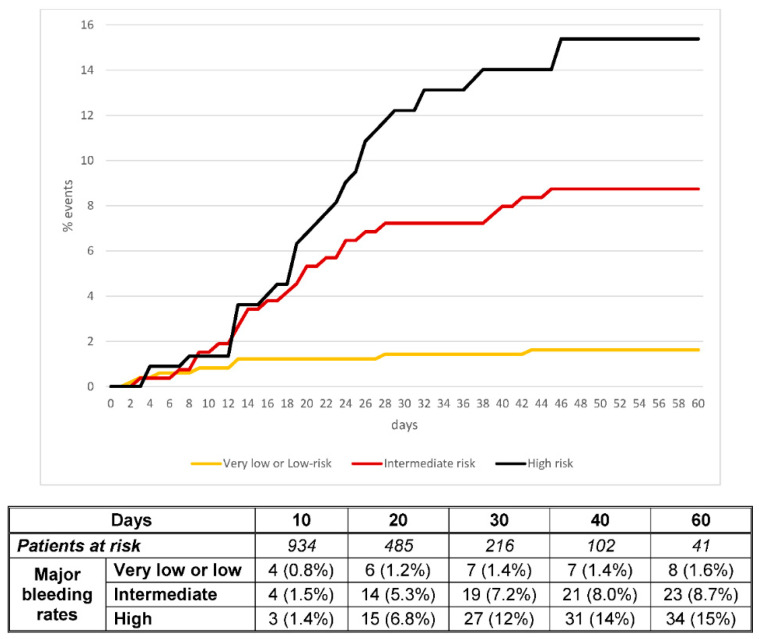
Cumulative incidence of major bleeding according to the prognostic score.

**Figure 2:**
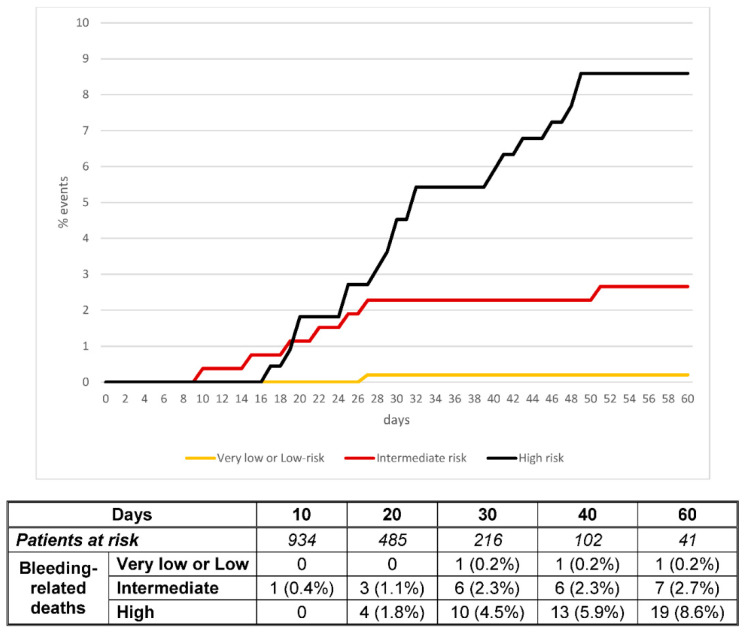
Cumulative incidence of bleeding-related death according to the prognostic score.

Using the prognostic score, 203 patients (21%) were considered to be at very low risk (0 points), 285 (29%) at low risk (1 point), 263 (27%) at intermediate risk (2 points) and 221 (23%) at high risk (3–4 points). Major bleeding occurred in 1.0%, 2.1%, 8.7% and 15.4% of the patients, respectively (Table 4). Non-major bleeding occurred in 0.5%, 3.5%, 9.5% and 14.2%, respectively. The proportion of patients who died within the first 30 days after major bleeding was: zero, 0.3%, 2.6% and 9.9%, respectively. The c-statistics were: 0.74 (95% CI: 0.68–0.79) for major bleeding, 0.73 (95% CI: 0.67–0.78) for non-major bleeding and 0.82 (95% CI: 0.76–0.87) for death within the first 30 days after bleeding. The score performed better in patients receiving standard- or intermediate-doses, and in those on other drugs than in those on therapeutic doses (Table 4). Figure 1 and Figure 2 show the different trends for cumulative incidence of major bleeding and death after bleeding, according to the prognostic score. The sensitivity, specificity, positive predictive value, and negative predictive value of the score for patients at high risk for bleeding were: 52.3 (95%CI: 40.2–64.5), 79.4 (95%CI: 76.7–82.0), 15.4 (95%CI: 10.6–20.1) and 95.9 (95%CI: 94.4–97.3), respectively.

## 4. Discussion

In the current study, one in every thirteen (6.7%) hospitalized patients for COVID-19 receiving VTE prophylaxis developed major bleeding during their hospital stay. This rate is similar to that reported in our previous study, and slightly (non-significantly) higher than the rates reported in a recent meta-analysis [4]. Interestingly, the mortality rate in patients suffering major bleeding was two-fold higher than in those who did not bleed (46% vs. 21%, respectively). Our prognostic score accurately identified patients at increased risk for major bleeding (c-statistics: 0.74; 95%CI: 0.68–0.79), but also for non-major bleeding (c-statistics: 0.73; 95%CI: 0.67–0.78) and death-related bleeding (c-statistics 0.82; 95%CI: 0.76–0.87). This is important since the score uses four items easily available at baseline (ICU admission, D-dimer levels, ferritin levels and high-dose prophylaxis) and might be of help to guide the prescription of the intensity of VTE prophylaxis.

In our previous study on 1965 patients hospitalized with COVID-19, the score showed a c-statistics of 0.74 (95%CI: 0.70–0.79) for major bleeding, and 0.72 (95%CI: 0.68–0.77) for non-major bleeding. The prognostic capacity of the score for death after bleeding was not evaluated [14]. In the current study, using a subsequent cohort of patients in the same registry, we obtained similar results. One of the limitations of the prior study was the exclusion of patients receiving standard doses of thromboprophylaxis. In the current study, we included 275 patients who received standard doses, confirming the relevance of the score for all treatment subgroups.

Bleeding is a major issue in hospitalized COVID-19 patients. Initial studies on the use of VTE prophylaxis in patients with COVID-19 focused on the high incidence of VTE, especially among patients admitted to the ICU [15,16,17,18,19]. Bleeding was considered a rare complication in the setting of COVID-19 at the beginning of the pandemics [20]. However, in a recent trial (INSPIRATION) including 562 patients with COVID-19 admitted to the ICU, major bleeding occurred in 2.5% of patients in the intermediate-dose group and in 1.4% of those in the standard-dose group [9]. Clinical trials (with rigorous inclusion and exclusion criteria) have reported a major bleeding rate of 0.9–8% [9,10,11,12], while observational studies and meta-analyses revealed higher rates of major bleeding in daily clinical practice, ranging from 2.7% to 21.6% [4,21,22,23,24,25].

An international, multiplatform, randomized clinical trial combining data from three trials (ACTIV-4a, REMAP-CAP and ATTACC) compared the use of therapeutic-dose heparin vs standard prophylaxis in hospitalized patients with COVID-19. In 2219 patients with critical illness, therapeutic-dose heparin was not associated with higher in-hospital survival or greater number of organ support-free days [10]. On the other hand, in 1098 noncritically ill patients, therapeutic-dose heparin did show a greater probability of survival with reduced use of cardiovascular or respiratory organ support [11]. Given these findings, the use of therapeutic doses of anticoagulation is expected to increase among COVID-19 patients with moderate disease, in whom the risk of bleeding is not considered high [26]. We humbly believe that our validated prognostic score may be useful to guide this decision.

Our study has certain limitations. First, the decision to prescribe each intensity of prophylaxis was decided by the attending physicians and protocols of each center. Second, the study was not aimed to ascertain the potentially beneficial effect of anticoagulation on survival or thrombotic events. Third, most patients were recruited during the first and second peak of COVID-19 outbreak, and all patients were included before the COVID-19 vaccine was available. Thus, our results might not be applicable to different populations. Fourth, some factors that could influence the development of bleeding, such as nosocomial infections, septic shock or oxygen support therapy have not been evaluated. On the other hand, our study has several strengths. This is the first study to validate a prognostic score to identify hospitalized patients with COVID-19 using real world data from a cohort of patients from different centers; thus, these results can have an impact in clinical practice. Second, the prognostic ability of the score was similar in patients receiving different types of anticoagulants at different doses. Third, in opposition to clinical trials, we had no exclusion criteria, thus our results reflect real world practice. Although the optimal dose of thromboprophylaxis in COVID-19 patients has not yet been established and several ongoing trials will help elucidate that question, our score can help to identify those patients where the bleeding risk is too high and more aggressive strategies of VTE prophylaxis should be discouraged.

In conclusion, in hospitalized COVID-19 patients, a simple prognostic score including four items easily available in clinical practice (elevated D-dimer, elevated ferritin, critical illness and therapeutic-intensity anticoagulation) may reliably identify those patients at increased risk for major bleeding, non-major bleeding or death within 30-days after bleeding. Since the use of high doses of heparin is expected to increase in hospitalized patients with COVID-19, the RIETE-BLEEDING score may be a helpful tool to assess bleeding risk.

## Figures and Tables

**Table 1:** Clinical characteristics of the patients, according to the use of different doses for VTE prophylaxis.

	N	Standard Doses	Intermediate Doses	Therapeutic Doses	Other Drugs
**Patients, N**	**972**	**280**	**412**	**157**	**123**
**Clinical characteristics,**					
Male gender	613 (63%)	173 (62%)	257 (62%)	112 (71%) *	71 (58%)
Age <70 years	562 (58%)	142 (51%)	266 (65%) ^‡^	98 (62%) *	56 (46%)
Body weight <70 kg	157 (22%)	54 (27%)	73 (25%)	16 (13%) ^†^	14 (17%)
Admitted in ICUs	464 (48%)	136 (49%)	177 (43%)	91 (58%)	60 (50%)
Recent major bleeding	10 (1.0%)	2 (0.7%)	5 (1.2%)	1 (0.6%)	2 (1.7%)
**Blood tests,**					
Anemia	287 (30%)	84 (30%)	106 (26%)	45 (29%)	52 (42%) *
Platelet count <100,000/μL	51 (5.3%)	15 (5.4%)	13 (3.2%)	6 (3.8%)	17 (14%) ^†^
Fibrinogen <1000 mg/dL	813 (89%)	234 (91%)	342 (87%)	134 (88%)	103 (95%)
Prothrombin time > 13.5 s	360 (38%)	88 (32%)	126 (31%)	61 (41%)	85 (73%) ^‡^
D-dimer > upper normal limit	845 (90%)	254 (93%)	362 (91%)	137 (91%)	92 (79%) ^‡^
D-dimer >10 × upper limit	336 (36%)	93 (34%)	130 (33%)	72 (48%) ^†^	41 (35%)
Ferritin >500 ng/mL (N = 809)	569 (70%)	169 (70%)	242 (69%)	99 (74%)	59 (68%)
CrCl < 60 mL/min	460 (47%)	143 (51%)	177 (43%) *	53 (34%) ^‡^	87 (71%) ^‡^
**Concomitant therapies,**					
Antiplatelet drugs	144 (15%)	39 (14%)	57 (14%)	18 (11%)	30 (25%) ^†^
**VTE prophylaxis,**					
Duration (median days, IQR)	14.7 ± 10.3	15.7 ± 10.5	14.9 ± 10.1	14.9 ± 10.4	11.8 ± 9.7 ^‡^
**Prognostic score,**					
Very low risk	203 (21%)	55 (20%)	111 (27%) *	0	37 (30%) *
Low risk	285 (29%)	96 (34%)	124 (30%)	34 (22%) ^†^	31 (25%)
Intermediate risk	263 (27%)	85 (30%)	106 (26%)	36 (23%)	36 (29%)
High risk	221 (23%)	44 (16%)	71 (17%)	87 (55%) ^‡^	19 (15%)
**Outcomes,**					
Non-major bleeding	67 (6.9%)	21 (7.5%)	23 (5.6%)	15 (9.6%)	8 (6.5%)
Major bleeding	65 (6.7%)	18 (6.4%)	21 (5.1%)	11 (7.0%)	15 (12%)
Bleeding-related death	30 (3.1%)	7 (2.5%)	10 (2.4%)	5 (3.2%)	8 (6.5%)
**All-cause mortality,**					
Yes	230 (24%)	53 (19%)	79 (19%)	46 (29%) *	52 (43%) ^‡^

Comparisons between patients with different doses of thromboprophylaxis vs. those receiving standard doses (reference subgroup): * *p* < 0.05; ^†^ *p* < 0.01; ^‡^ *p* < 0.001. **Abbreviations*:*** SD, standard deviation; ICUs, intensive care units; IL-6, interleukine-6; Sec, seconds; CrCl, creatinine clearance; IQR, inter-quartile range.

**Table 2:** Baseline characteristics of the patients, according to the development of bleeding events.

	Major Bleeding	Non-Major Bleeding	No Bleeding	All Patients
**Patients, N**	**65**	**67**	**840**	**972**
**Clinical characteristics,**				
Male gender	47 (72%)	45 (67%)	521 (62%)	613 (63%)
Age <70 years	37 (57%)	46 (69%)	479 (57%)	562 (58%)
Body weight <70 kg	7 (13%)	11 (22%)	139 (23%)	157 (22%)
Admitted in ICUs	54 (83%) ^‡^	59 (88%) ^‡^	351 (42%)	464 (48%)
Recent major bleeding	0	1 (1.5%)	9 (1.1%)	10 (1.0%)
**Blood tests,**				
Anemia	17 (26%)	28 (42%) *	242 (29%)	287 (30%)
Platelet count <100,000/μL	4 (6.2%)	4 (6.0%)	43 (5.1%)	51 (5.3%)
Fibrinogen <1000 mg/dL	52 (84%)	55 (89%)	706 (90%)	813 (89%)
Prothrombin time >13.5 s	21 (32%)	22 (33%)	317 (39%)	360 (38%)
D-dimer > upper normal limit	63 (98%) ^†^	62 (97%)	720 (89%)	845 (90%)
D-dimer >10 × upper limit	46 (72%) ^‡^	37 (58%) ^‡^	253 (31%)	336 (36%)
Ferritin >500 ng/mL (N = 809)	48 (84%) ^†^	53 (80%) *	463 (67%)	564 (70%)
CrCl <60 mL/min	33 (51%)	27 (40%)	400 (48%)	460 (47%)
**Concomitant therapies,**				
Antiplatelets	15 (23%) *	14 (21%)	115 (14%)	144 (15%)
**VTE prophylaxis,**				
Standard doses	18 (28%)	21 (31%)	241 (29%)	280 (29%)
Intermediate doses	21 (32%)	23 (34%)	368 (44%)	412 (42%)
Therapeutic doses	11 (17%)	15 (22%)	131 (16%)	157 (16%)
Other drugs	15 (23%) *	8 (12%)	100 (12%)	123 (13%)
Duration (median days, IQR)	16 (10–26)	15 (10–23)	12 (7–18)	12 (7–19)
Duration (>10 days)	46 (71%) *	48 (72%) *	473 (56%)	567 (58%)
**30-day mortality,**				
Yes	30 (46%) ^‡^	22 (33%) *	178 (21%)	230 (24%)

Comparisons between patients with- vs. without bleeding: * *p* < 0.05; ^†^ *p* < 0.01; ^‡^ *p* < 0.001. **Abbreviations:** SD, standard deviation; ICUs, intensive care units; IL-6, interleukine-6; CrCl, creatinine clearance; IQR, inter-quartile range.

**Table 3:** Sites of bleeding.

	Major Bleeding	Bleeding-Related Death	Non-Major Bleeding	All Patients
**Patients, N**	**65**	**30**	**67**	**132**
Orotracheal	14 (22%)	8 (27%)	15 (22%)	29 (22%)
Gastrointestinal	18 (28%)	7 (23%)	7 (10%)	25 (19%)
Hematoma	10 (15%)	5 (17%)	7 (10%)	17 (13%)
Genitourinary	4 (6.2%)	1 (3.3%)	4 (6.0%)	8 (6.1%)
Alveolar	4 (6.2%)	1 (3.3%)	4 (6.0%)	8 (6.1%)
Abdominal	5 (7.7%)	1 (3.3%)	0	5 (3.8%)
Muscular	4 (6.2%)	1 (3.3%)	1 (1.5%)	5 (3.8%)
Intracranial	3 (4.6%)	1 (3.3%)	0	3 (2.3%)
Other	3 (4.6%)	5 (17%)	29 (43%)	32 (24%)

**Table 4:** Proportion of patients developing bleeding complications according to the prognostic score. The score assigns 1 point for each of the following: ICU admission, D-dimer levels > 10 times over the upper limit, ferritin levels > 500 ng/mL and use of therapeutic anticoagulation.

	Patients, N	Major Bleeding	Non-Major Bleeding	Bleeding-Related Death
**Estimated risk**				
**All patients**	**972**	**65 (6.7%)**	**67 (6.9%)**	**30 (3.09%)**
Very low risk (0 points)	203	2 (1.0%)	1 (0.5%)	0
Low risk (1 point)	285	6 (2.1%)	10 (3.5%)	1 (0.35%)
Intermediate risk (2 points)	263	23 (8.7%)	25 (9.5%)	7 (2.6%)
High risk (3–4 points)	221	34 (15.4%)	31 (14.2%)	22 (9.9%)
**C-statistics (95%CI)**				
**All patients**	**972**	**0.74 (0.68–0.79)**	**0.73 (0.67–0.78)**	**0.82 (0.76–0.87)**
Standard doses	280	0.72 (0.60–0.84)	0.72 (0.63–0.82)	0.85 (0.70–1.00)
Intermediate doses	412	0.76 (0.68–0.86)	0.72 (0.63–0.82)	0.84 (0.76–0.91)
Therapeutic doses	157	0.66 (0.51–0.80)	0.76 (0.63–0.88)	0.79 (0.66–0.92)
Other drugs	123	0.82 (0.72–0.92)	0.74 (0.61–0.86)	0.86 (0.77–0.95)

**Abbreviations:** CI, confidence intervals.

## Data Availability

Data available on request due to privacy restrictions.

## Supplementary Materials

The following are available online at https://www.mdpi.com/article/10.3390/v13112278/s1, Table S1: Proportions of patients receiving standard-, intermediate- or therapeutic doses of anticoagulants for thromboprophylaxis in the participating centers; Table S2: Treatments used for thromboprophylaxis; Table S3: STROBE statement (parts I and II).

## Appendix A

Coordinator of the RIETE-BLEEDING Registry: Manuel Monreal.

RIETE-BLEEDING Registry Coordinating Center: S & H Medical Science Service.

Members of the RIETE-BLEEDING Group:

**SPAIN**: Adarraga D, Aibar J, Baeza C, Ballaz A, Barba R, Blanco-Molina A, Botella E, Criado J, de Ancos C, del Toro J, Demelo-Rodríguez P, Díaz-Brasero AM, Díaz-Pestano MM, Farfán-Sedano AI, Fernández-Capitán C, Fidalgo A, Flores K, Gabara C, Galeano-Valle F, Gavín-Sebastián O, Gil-Díaz A, Jaras MJ, Jara-Palomares L, Jiménez R, Lainez-Justo S, Latorre A, Lecumberri R, Llamas P, Lobo JL, López-Jiménez L, Loureiro B, Madridano O, Mancebo-Plaza AB, Martín del Pozo M, Monreal M, Muñoz-Rivas N, Núñez-Fernández MJ, Olivera PE, Ordieres-Ortega L, Padín-Paz EM, Pedrajas JM, Quintana-Díaz M, Ríos-Prego M, Rodríguez-Chiaradía DA, Ruiz-Artacho P, Sigüenza P, Suriñach JM, Trujillo-Santos J, Zamora C, **ITALY**: Bucherini E, Di Micco P, Imbalzano E, Siniscalchi C, **R****EPUBLIC OF MACEDONIA**: Bosevski M, Stevanovic M, **USA**: Paz-Rios L, Weinberg I.
