# Supplementary material for: Validation of a Prognostic Score to Identify Hospitalized Patients with COVID-19 at Increased Risk for Bleeding
Source: Viruses. 2021 Nov 15;13(11):2278. doi: 10.3390/v13112278 (PMC8621368; doi:10.3390/v13112278)
Supplement: Supplementary file 1 [file viruses-13-02278-s001.zip › viruses-1436051-supplementary.pdf]

Supplementary Table I. Proportions of patients receiving standard-, intermediate- or therapeutic doses of anticoagulants for thromboprophylaxis in the participating centers.

Hospitals	N	Standard doses	Intermediate doses	Therapeutic doses	Other drugs
Patients, N	***972***	***280***	***412***	***280***	***123***
Hospital Universitario de Fuenlabrada	144	20 (14%)	59 (41%)	31 (21%)	34 (24%)
Hospital Universitario La Paz, Madrid	127	26 (20%)	66 (52%)	34 (27%)	1 (0.8%)
Hospital Germans Trias i Pujol, Badalona	124	41 (33%)	59 (48%)	14 (11%)	10 (8.1%)
Hospital Fundación Jiménez Díaz, Madrid	111	51 (46%)	52 (47%)	8 (7.2%)	0
Hospital Universitario de Gran Canaria	66	27 (41%)	21 (32%)	9 (14%)	9 (14%)
Complejo Hospitalario de Pontevedra	41	12 (29%)	26 (63%)	3 (7.3%)	0
Massachusetts General Hospital, US	41	6 (15%)	6 (15%)	0	29 (71%)
Hospital de Cantoblanco, Madrid	37	15 (41%)	19 (51%)	3 (8.1%)	0
Hospital Gregorio Marañón, Madrid	35	6 (17%)	6 (17%)	4 (11%)	19 (54%)
Hospital Universitario Vall d'Hebron, Barcelona	35	16 (46%)	2 (5.7%)	16 (46%)	1 (2.9%)
Evanston NorthShore University HealthSystem	31	0	19 (61%)	6 (19%)	6 (19%)
Hospital Universitari Sagrat Cor, Barcelona	27	9 (33%)	16 (59%)	2 (7.4%)	0
Hospital General Universitario de Elche	23	5 (22%)	7 (30%)	11 (48%)	0
Hospital de Galdakao, Bizkaia	22	7 (32%)	14 (64%)	1 (4.5%)	0
Hospital Universitario Txagorritxu, Vitoria	20	1 (5%)	19 (95%)	0	0
Hospital del Mar, Barcelona	11	2 (18%)	6 (55%)	3 (27%)	0
Clínica Universidad de Navarra	11	8 (73%)	1 (9.1%)	2 (18%)	0
Azienda Ospedaliera Universitaria di Parma	10	4 (40%)	4 (40%)	2 (20%)	0
Hospital Clínico San Carlos, Madrid	9	1 (11%)	1 (11%)	2 (22%)	5 (56%)
Hospital Universitario Infanta Sofía, Madrid	8	4 (50%)	1 (12%)	1 (12%)	2 (25%)
Hospital Universitario de Salamanca	8	2 (25%)	3 (37%)	2 (25%)	1 (12%)
Hospital Universitario Clínic de Barcelona	8	4 (50%)	2 (25%)	1 (12%)	1 (12%)
Hospital Universitario Rey Juan Carlos, Madrid	8	2 (25%)	0	1 (12%)	5 (62%)
Hospital Universitario de Guadalajara	5	3 (60%)	2 (40%)	0	0
Hospital Universitario Reina Sofía, Córdoba	4	3 (75%)	1 (25%)	0	0
Ospedale Buon Consiglio Fatebenefratelli, Napoli	3	3 (100%)	0	0	0
Hospital Universitario Santa Lucía, Cartagena	1	1 (100%)	0	0	0
Hospital Doctor José Molina Orosa	1	0	0	1 (100%)	0
A.O.U Policlinico “G. Martino”	1	1 (100%)	0	0	0

Supplementary Table II. Treatments used for thromboprophylaxis.

	**Standard doses**	**Intermediate doses**	**Therapeutic doses**	**Other drugs**
**Standard doses,**	***280***	-	-	-
Enoxaparin 40 mg daily	184 (66%)	-	-	-
Bemiparin 2,500-3,500 IU daily	59 (21%)	-	-	-
Biosimilars of enoxaparin 40 mg daily	15 (5.4%)	-	-	-
Tinzaparin 2,500-4,500 IU daily	10 (3.6%)	-	-	-
Apixaban 5 mg daily	8 (2.9%)	-	-	-
Fondaparinux 2.5 mg daily	5 (1.8%)	-	-	-
				-
**Intermediate doses,**	-	***412***	-	-
Enoxaparin 60-100 mg daily	-	334 (81%)	-	-
Biosimilars of enoxaparin 60- 100 mg daily	-	38 (9.2%)	-	-
Bemiparin 5,000-7,500 IU daily	-	27 (6.6%)	-	-
Apixaban 10 mg daily	-	6 (1.5%)	-	-
Fondaparinux 5.0 mg daily	-	4 (1.0%)	-	-
Edoxaban 30 mg daily	-	2 (0.5%)	-	-
Rivaroxaban 10 mg daily	-	1 (0.2%)	-	-
Therapeutic doses,			***157***	-
Enoxaparin 120- 220 mg daily	-	-	121 (77%)	-
Bemiparin 10,000-12,500 IU daily	-	-	13 (8.3%)	-
Biosimilars enoxaparin 120- 220 mg daily	-	-	6 (3.8%)	-
Tinzaparin 10,000-14,000 IU daily	-	-	6 (3.8%)	-
Fondaparinux 7.5 mg daily	-	-	6 (3.8%)	-
Rivaroxaban 20 mg daily	-	-	2 (1.3%)	-
Dabigatran 220 mg daily	-	-	2 (1.3%)	-
Edoxaban 60 mg daily	-	-	1 (0.6%)	-
**Other drugs,**	-	-	-	***123***
Unfractionated heparin	-	-	-	68 (55%)
Vitamin K antagonists	-	-	-	55 (45%)

Abbreviations: IU, international units.

Supplementary Table III. STROBE statement (part I)

	Item No	Recommendation
**Title and abstract**	1	(*a*) Indicate the study's design with a commonly used term in the title or the abstract PAGE 1
		(*b*) Provide in the abstract an informative and balanced summary of what was done and what was found PAGE 2
**Introduction**		
Background/rationale	2	Explain the scientific background and rationale for the investigation being reported PAGES 3-4
Objectives	3	State specific objectives, including any prespecified hypotheses PAGE 4
**Methods**		
Study design	4	Present key elements of study design early in the paper PAGES 4-5
Setting	5	Describe the setting, locations, and relevant dates, including periods of recruitment, exposure, follow-up, and data collection PAGES 4-5
Participants	6	(*a*) *Cohort study*—Give the eligibility criteria, and the sources and methods of selection of participants. Describe methods of follow-up PAGES 4-5
Variables	7	Clearly define all outcomes, exposures, predictors, potential confounders, and effect modifiers. Give diagnostic criteria, if applicable PAGES 5-6
Data sources/ measurement	8*	For each variable of interest, give sources of data and details of methods of assessment (measurement). Describe comparability of assessment methods if there is more than one group PAGES 5-6
Bias	9	Describe any efforts to address potential sources of bias PAGES 4-6
6Study size	10	Explain how the study size was arrived at PAGE 4
Quantitative variables	11	Explain how quantitative variables were handled in the analyses. If applicable, describe which groupings were chosen and why PAGE 6
Statistical methods	12	(*a*) Describe all statistical methods, including those used to control for confounding PAGE 6
		(*b*) Describe any methods used to examine subgroups and interactions PAGE 6
		(*c*) Explain how missing data were addressed PAGE 6
		(*d*) *Cohort study*—If applicable, explain how loss to follow-up was addressed PAGE 6
		(*e*) Describe any sensitivity analyses

Supplementary Table IV. STROBE statement (part II)

**Results**		
Participants	13*	(a) Report numbers of individuals at each stage of study—eg numbers potentially eligible, examined for eligibility, confirmed eligible, included in the study, completing follow-up, and analysed PAGE 7 and Supplementary Table I
		(b) Give reasons for non-participation at each stage
		(c) Consider use of a flow diagram
Descriptive data	14*	(a) Give characteristics of study participants (eg demographic, clinical, social) and information on exposures and potential confounders Table I
		(b) Indicate number of participants with missing data for each variable of interest not reported
		(c) *Cohort study*—Summarise follow-up time (eg, average and total amount) Table I
Outcome data	15*	*Cohort study*—Report numbers of outcome events or summary measures over time Table I
Main results	16	(*a*) Give unadjusted estimates and, if applicable, confounder-adjusted estimates and their precision (eg, 95% confidence interval). Make clear which confounders were adjusted for and why they were included Table II
		(*b*) Report category boundaries when continuous variables were categorized
		(*c*) If relevant, consider translating estimates of relative risk into absolute risk for a meaningful time period
Other analyses	17	Report other analyses done—eg analyses of subgroups and interactions, and sensitivity analyses Table IV, and figures 1 and 2.
**Discussion**		
Key results	18	Summarise key results with reference to study objectives PAGE 8
Limitations	19	Discuss limitations of the study, taking into account sources of potential bias or imprecision. Discuss both direction and magnitude of any potential bias PAGE 9
Interpretation	20	Give a cautious overall interpretation of results considering objectives, limitations, multiplicity of analyses, results from similar studies, and other relevant evidence PAGE 10
Generalisability	21	Discuss the generalisability (external validity) of the study results PAGE 10
**Other information**		
Funding	22	Give the source of funding and the role of the funders for the present study and, if applicable, for the original study on which the present article is based. PAGE 10.
